# Cisplatin and paclitaxel co-delivered by folate-decorated lipid carriers for the treatment of head and neck cancer

**DOI:** 10.1080/10717544.2016.1236849

**Published:** 2017-05-11

**Authors:** Jiying Yang, Zengjuan Ju, Shufang Dong

**Affiliations:** Department of Pharmacy, Linyi People’s Hospital, Linyi, Shandong Province, PR China

**Keywords:** Cisplatin, co-delivery, head and neck cancer, lipid carriers, paclitaxel

## Abstract

*Context*: For head and neck cancer therapy, co-delivery of two drugs, cisplatin (DDP) plus paclitaxel (PTX), are more effective than single drug therapy. Lipid carriers are promising drug carriers for anti-cancer delivery.

*Objective*: The aim of this study is to construct a folate (FA) decorated nanostructured lipid carriers (NLCs) as nanocarriers for DDP and PTX delivery.

*Materials and methods*: In this study, DDP and PTX were incorporated into NLCs. Folate-PEG-DSPE (FA-PEG-DSPE) was synthesized and decorated the drugs-loaded NLCs (FA-DDP/PTX NLCs). Their average size, zeta potential, drug encapsulation efficiency, drug loading capacity, and *in vitro* drug release were evaluated. Head and neck cancer cells (FaDu cells) were used for the testing of *in vitro* cytotoxicity, and *in vivo* transfection efficiency of NLC was evaluated on mice bearing FaDu cells model.

*Results*: The size of FA-DDP/PTX NLCs was around 127 nm, with a positive zeta potential of 26.7 mV. FA-DDP/PTX NLCs showed the highest cytotoxicity and synergistic effect of two drugs in head and neck cancer cells (FaDu cells) *in vitro*. The *in vivo* study revealed the greatest anti-tumor activity than all the other formulations in murine-bearing head and neck cancer model.

*Discussion and conclusion*: FA-DDP/PTX NLCs effectively improves anticancer efficiency for head and neck cancer *in vitro* and *in vivo*. The constructed NLCs could be used as a novel carrier to co-delivery DDP and PTX for head and neck cancer therapy.

## Introduction

Head and neck cancer is the sixth most common cancer worldwide, accounting for 5–6% of all cancer cases, with an estimated 900 000 new cases and 350 000 mortalities every year (Wu & Zhou, 2015). Almost all these cancers are head and neck squamous cell carcinomas (HNSCC); and most HNSCC patients present with advanced stage disease (stage III/IV) (Soundararajan et al., 2009). The clinical outcomes and overall survival rates for advanced HNSCC have not improved significantly over the past two decades despite advancements in surgery and treatment (Duan et al., 2012). Therefore, it is urgent to design novel therapies to achieve a more favorable clinical outcome and reduce treatment morbidity (Wilken et al., 2011).

Based on the NCCN practice guidelines, standard treatment regimens for head and neck cancer depend on the stage of the disease. Advanced cancers often require multi-modality therapy with surgery, radiation, and chemotherapy (Posner, 2010). Platinum-based agents form the backbone of the standard chemotherapeutic regimens for head and neck cancer; and active combination regimens include cisplatin (DDP) plus 5-FU, or taxane, or cetuximab (Pfister et al., 2014). While DDP plus paclitaxel (PTX) are more effective than single drug therapy, they are known to possess adverse reaction profiles. DDP induces renal toxicity; and PTX has shown dose-limiting hematological toxicity (e.g. neutropenia), sensory, etc. (Rowinsky et al., 1993; Oberoi et al., 2013).

Lipid carriers such as liposomes (Doddapaneni et al., 2016), solid lipid nanoparticles (Jia et al., 2016), lipid–polymer hybrid nanoparticles (Fang et al., 2010), and nanostructured lipid carriers (NLCs) (Alam et al., 2013) have been used to encapsulate drugs for the cancer therapy. NLCs, formulated with biocompatible solid and liquid lipids, are an improved generation of SLNs, providing a delivery system for various active drugs with controlled-release characteristic (Song et al., 2016). It has been well documented that NLCs were developed for overcoming some SLN limitations to their highly ordered crystalline structure. NLCs act as a new type of lipid drug delivery system, offering the advantages of improved drug loading and sustained release (Zhang et al., 2014).

As a natural material, FA has several advantages as a potential targeting agent, including lower molecular weight and immunogenicity than most antibodies, relatively high stability, and ease of synthesis (Wang et al., 2009). Importantly, the folate receptor (FR) is highly expressed in head and neck cancers (Dosio et al., 2009; Ward et al., 2011; Xie et al., 2013). Conversely, normal tissues, most notably bone marrow tissue, lack FR expression, making folate an excellent tumor-targeting moiety (Saba et al., 2009). In this study, FA was chosen as the ligand to modify DDP/PTX NLCs. In the present study, DDP/PTX NLCs were prepared by the nanoprecipitation technology. FA-PEG-DSPE was synthesized and modified onto the surface of drugs loaded NLCs. The *in vitro* cytotoxicity studies of different formulations were evaluated on head and neck cancer cells (FaDu cells). *In vivo* anti-tumor effects were observed on the murine bearing FaDu cells model.

## Materials and methods

### Materials

PEG-DSPE was provided by CordenPharma International (Plankstadt, Germany). Folate, cholesterol, and (3-[4,5-dimehyl-2-thiazolyl]-2,5-diphenyl-2*H*-tetrazolium bromide (MTT) were purchased from Sigma-Aldrich Co., Ltd (St Louis, MO). Polyoxyl castor oil (Cremophor ELP) was donated by BASF (Ludwigshafen, Germany). Glyceryl behenate (COMPRITOL 888 ATO) was generously provided by Gattefossé China (Shanghai, China). Cetyltrimethyl ammonium bromide (CTAB) was purchased from Yixing Kailida Chemical Co., Ltd (Wuxi, China). Cisplatin (DDP) was purchased from Zhejiang Haiqiang Chemicals Co., Ltd (Hangzhou, China). Paclitaxel (PTX) was obtained from Ji’nan Haohua Industry Co., Ltd (Ji’nan, China). FaDu cells were obtained from the American type culture collection (Manassas, VA). All other chemicals were of analytical grade or higher.

### Animals

BALB/c mice (6–8 weeks old, 18–22 g weight) were purchased from SLAC Laboratory Animal Co., Ltd (Shanghai, China), and housed under standard laboratory conditions. All animal experiments complied with the Animal Management Rules of the Ministry of Health of the People’s Republic of China.

### Synthesis of FA-PEG-DSPE

FA-PEG-DSPE was synthesized by covalent coupling of FA with NH_2_-PEG_2000_-DSPE (Gabizon et al., 1999; Tomasina et al., 2013). Briefly, folate (0.15 mmol) was dissolved in DMSO (3 mL). Amino-PEG2000-DSPE (0.1 mmol) and pyridine (1.5 mL) were added to the FA-DMSO solution, followed by dicyclohexylcarbodiimide (0.45 mmol). The reaction was continuously stirred for 12 h at room temperature. Pyridine was removed by rotary evaporation. Water (50 mL) was added to the reaction mixture. The solution was filtered and the filtrate was dialyzed against Milli-Q water. FA-PEG-DSPE was obtained by lyophilization. The product has a yield of 88.7%.

### Preparation of DDP/PTX NLCs

The lipid phase was composed of COMPRITOL® 888 ATO, olive oil, and Cremophor ELP at a ratio of 2:1:1 and was melted by heating to approximately 70 °C (Rahman et al., 2013). A cold aqueous phase was prepared by dissolving CTAB 0.2 g, Tween-80 1.0 g, in double-distilled water and made up to 100 mL. This aqueous surfactant solution was then heated with stirring to the same temperature as the lipid matrices. The lipid phase was rapidly injected into the hot surfactant solution using a mechanical agitate (1000 rpm, 1 h). The NLCs dispersions were obtained by dispersing warm o/w nanoemulsion into icy distilled water (0 °C).

Single drug (DDP or PTX)-loaded NLCs and drug free NLCs were prepared by the same method with the presence of one drug or no drug, named DDP NLCs, PTX NLCs, and NLCs.

### Decoration of DDP/PTX NLCs with FA-PEG-DSPE

**Figure 1.**
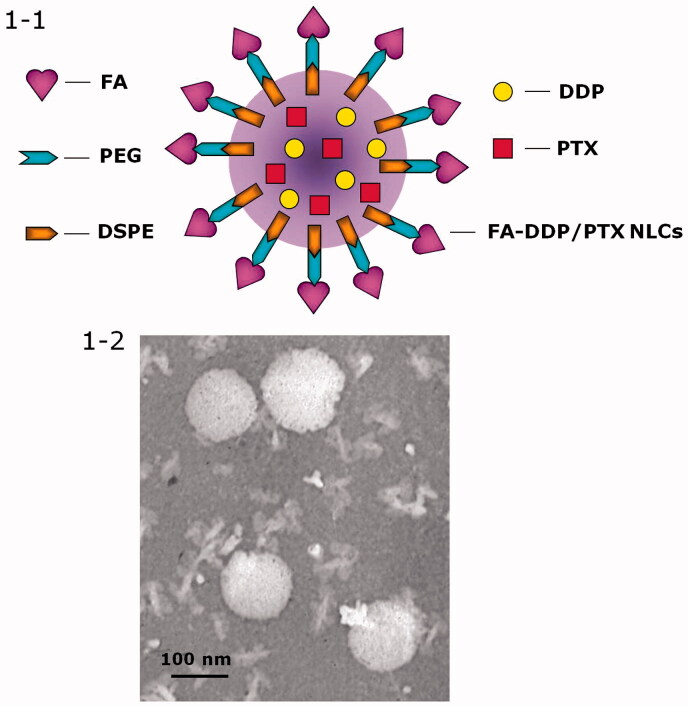
(1) Scheme graph of the construction of FA-DDP/PTX NLCs; (2) TEM image of FA-DDP/PTX NLCs.

Decoration of DDP/PTX NLCs with FA-PEG-DSPE was carried out by electrostatic attraction and the decoration ratio was optimized (Han et al., 2014). Briefly, the FA-PEG-DSPE ligands were dissolved in 40 ml of PBS. The dispersion was added dropwise into 60 mL of DDP/PTX NLCs under the stirring at 400 rpm at room temperature. 2 h of agitation was followed to complete the decoration. To select the suitable ratio of FA-PEG-DSPE ligands used in the formulation, FA-PEG-DSPE solution containing 0.1, 0.2, 0.3, 0.4, and 0.5 g of FA-PEG-DSPE were prepared separately. Zeta potential and size of the decorated DDP/PTX NLCs were measured. The FA-PEG-DSPE weight ratio was optimized by measuring the change in zeta potential and size. The obtained complexes was resuspended in Milli-Q water and filtered through a membrane with 0.45 μm pore size to obtain FA-PEG-DSPE decorated DDP/PTX NLCs (FA-DDP/PTX NLCs, Figure 1(1)).

### Characterization

Characterization of FA-DDP/PTX NLCs, DDP/PTX NLCs, DDP NLCs, PTX NLCs, and NLCs were carried out as follows.

#### Particle morphology, size and zeta potential

Particle morphology of FA-DDP/PTX NLCs was observed by a Transmission Electron Microscope (TEM, Hitachi, Tokyo, Japan). Diluted FA-DDP/PTX NLCs were placed on a carbon-coated copper grid, negatively stained with 2% phosphotungstic acid, and then observed with TEM.

The mean particle size, polydispersity index (PDI), and zeta potential were analyzed by photon correlation spectroscopy (PCS), with a Zetasizer 3000 (Malvern Instruments, Malvern, England). The average particle size was expressed as volume mean diameter (Wang et al., 2012).

#### Drug encapsulation efficiency and drug-loading capacity

The drug encapsulation efficiency (DEE) and drug-loading capacity (DLC) of DDP or PTX were measured differently. For DDP, DEE and DLC were determined using the UV-visible spectrophotometric method of DDP with *o*-phenylenediamine (Vhora et al., 2014). In brief, NLCs formulations were heated at 90 °C for 30 min with *o*-phenylenediamine solution in dimethylformamide (DMF). The dilutions were made with DMF-water mixture (7:3, v/v, pH 6.2 adjusted with 0.1 N HCl), and the reaction product was estimated at 705 nm on the UV-visible spectrophotometer (UV-1800, Shimadzu, Tokyo, Japan).

For PTX, DEE and DLC were estimated by a subtraction method (Shao et al., 2015). Briefly, the obtained NLCs were precipitated by the pH adjustment. After the centrifugation, the NLCs precipitate was obtained and the drug content in the supernatant was measured by HPLC (Agilent 1260 series, Richardson, TX). Chromatographic separations were carried out using the Inertsil® ODS-3V (250 mm × 4.6 mm). Mobile phase consisted of a mixture of acetonitrile and water (50:50, v/v). The flow rate was kept at 1.0 ml/min and system was maintained at 35 °C, the detection was carried out at 227 nm. The injection volume was 20 μL.

DEE were calculated as follows: DEE (%) = weight of (total drug − free drug)/weight of total drug^ ^× 100.

DLC were calculated as follows: DLC (%) = weight of (total drug − free drug)/weight of total drug and lipid carriers × 100.

### Serum stability

Serum stability of FA-DDP/PTX NLCs, DDP/PTX NLCs, DDP NLCs, and PTX NLCs was evaluated in phosphate buffers (PBS) solution containing 10% fetal bovine serum (FBS, v/v) at 37 °C for 24 h, separately (Golla et al., 2013). At 0, 1, 2, 4, 8, 12, and 24 h, 1 mL of each sample was diluted with 2 mL THF and the mixture was bath sonicated for 5 min, followed by centrifugation at 10 000 rpm for 5 min. The variation trends of the particle size and DEE were calculated by the same method mentioned in section Drug encapsulation efficiency and drug-loading capacity.

### *In vitro* drug release study

*In vitro* DDP or PTX released from FA-DDP/PTX NLCs, DDP/PTX NLCs, DDP NLCs, PTX NLCs were measured by the dialysis method (Sonali et al., 2016). Different formulations were placed in the dialysis bag separately. Then, the bag was incubated with 50 mL release medium (0.1% Tween 80 in PBS, pH 7.4 and 5.0, respectively). The medium (1 mL) was collected at predetermined time points and replaced with 50 mL of fresh medium. The concentrations of released DDP or PTX were determined by the methods of section “Characterization”.

### *In vitro* cytotoxic study

#### Cells culture

The head and neck cancer cells (FaDu cells) were maintained in Dulbecco’s modified Eagle’s medium (DMEM) supplemented with 4.5 g/l glucose and 10% fetal bovine serum (Invitrogen, Carlsbad, CA), 4 mM glutamine, 100 IU/ml penicillin, and 100 μg/ml streptomycin (Pereira Ade et al., 2013). Five days prior to the assay 10,000 cells/well were plated into 96-well tissue culture plates and cultured at 37 °C in a 5% CO_2_ atmosphere.

#### Cytotoxicity assay

The cytotoxicity was evaluated by MTT assay (Wang et al., 2007). The formulations with varying concentrations were added to each well. The plates were then returned to the incubators. After 24 h, aliquots of MTT solution (20 ml) were added into each well after the designated period. The plates were then returned to the incubator. After 3 h of incubation, the growth medium in each well was removed, and 150 ml of dimethylsulfoxide (DMSO) were added to each well to dissolve the internalized purple formazan crystals. An aliquot of 100 ml was taken from each well, and transferred to a new 96-well plate. The plates were then assayed at 550 and 690 nm using a microplate reader. The absorbance readings of the formazan crystals were taken to be that at 550 nm subtracted by that at 690 nm. The sodium acetate buffer of an equivalent volume was used as the negative control. The results were expressed as a percentage of the absorbance of the negative control. Half maximal inhibitory concentration (IC_50_) values of samples were calculated. Tumor cell proliferation inhibition behavior of FA-DDP/PTX NLCs was evaluated. The Combination Index (CI) was measured according to the Chou and Talalay’s method (Lv et al., 2014). To distinguish synergistic, additive, or antagonistic cytotoxic effects, the following equation was used: CI*_x_* = (D)_1_/(D*_x_*)_1_ + (D)_2_/(D*_x_*)_2_. (D*_x_*)_1_ and (D*_x_*)_2_ represent the IC*_x_* value of DDP and PTX alone, respectively. (D)_1_ and (D)_2_ represent the concentration of DDP and PTX in the combination system at the IC*_x_* value. CI > 1 represents antagonism, CI = 1 represents additive, and CI < 1 represents synergism. In this study, IC_50_ value (inhibitory concentration to produce 50% cell death) was applied.

### *In vivo* tissue distribution study

The head and neck cancer cells (FaDu cells) were transplanted subcutaneously into the armpits of the mice (Wang et al., 2016). One day following transplantation, the mice were randomly allocated to either the control (vehicle control, received PBS) or treatment groups, with 10 mice in each group. FA-DDP/PTX NLCs was administered intravenously every 3 days until 3 weeks. All efforts were made to minimize the suffering of the animals and to reduce the number of animals used and the mice were sacrificed by cervical dislocation. The *in vivo* tissue distribution study was investigated at 10 min, 1 h, 8 h, 24 h, and 48 h after intravenous injection. At predetermined time intervals, mice were sacrificed and the tumor, heart, liver, spleen, lung, and kidney of mice were collected. Tissues were initially weighed and homogenized with physiological saline to determine the amount of DDP or PTX in each tissue. The concentrations of released DDP or PTX were determined the same as described in section Drug encapsulation efficiency and drug-loading capacity.

### *In vivo* anticancer evaluation

FaDu cells were transplanted subcutaneously into the armpits of the mice. One day following transplantation, the mice were randomly allocated to either the control or treatment groups, with 10 mice in each group. The different formulations were administered intravenously every 3 d until 3 weeks. After the mice were sacrificed, the solid tumors were separated. The volumes of the solid tumor were measured with a digital caliper every 3 d and were calculated by the formula: Tumor volume = (*W*^2 ^× *L*)/2, where *W* is the tumor measurement at the widest point and *L* the tumor dimension at the longest point (Zhang et al., 2016). Tumor weights were measured and the tumor growth inhibition ratio (TGI) was calculated as follows: TGI (%) = (weight of control − weight of treated)/weight of control × 100. In order to evaluate the systemic toxicity of different systems, the body weight variation was calculated.

## Results

### Decoration ratio determination

Table 1 shows the change of size and potential during the decoration process. The change of zeta potential was stable after the weight of FA-PEG-DSPE increased above 0.3 g. The size of FA-DDP/PTX NLCs was slowly increased until the weight of FA-PEG-DSPE increased to 0.4 g, however, the suddenly increase of size was observed when FA-PEG-DSPE was 0.5 g. So the decoration ratio was determined as 0.3 g of FA-PEG-DSPE dissolved in 40 ml of PBS to get the FA-PEG-DSPE solution in the preparation process of FA-DDP/PTX NLCs.

**Table 1.** Decoration ratio determination.

Weight of ligands (g)	0.1	0.2	0.3	0.4	0.5
Zeta potential (mV)	+36.3 ± 2.6	+ 31.9 ± 2.3	+ 26.6 ± 1.9	+ 26.4 ± 2.1	+ 26.8 ± 2.4
Particle size (nm)	118.3 ± 2.9	121.3 ± 3.1	126.9 ± 3.2	128.4 ± 3.6	618.3 ± 54.6

### Characterization

The NLCs or NLCs loaded with one or two drugs exhibit particle size of around 100 nm (Table 2). This could be the evidence that loading of drugs would not enlarge the particle size. After the decoration of FA-PEG-DSPE, the particles size of FA-DDP/PTX NLCs reached about 127 nm. The coating of ligands increased the size of the carriers. The zeta potential of FA-DDP/PTX NLCs goes down from 43.7 mV to 26.7 mV after decoration. FA-DDP/PTX NLCs display a spherical morphology with some white coats on the surface (Figure 1(2)).

**Figure 2.**
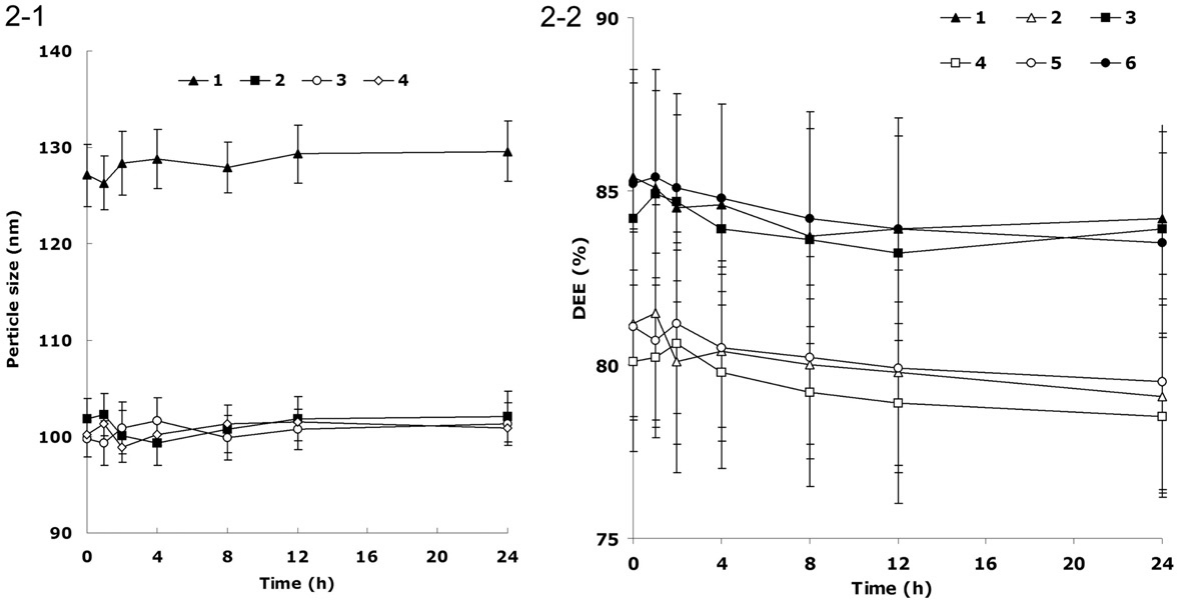
(1) Changes in size in the presence of serum: (1) FA-DDP/PTX NLCs, (2) DDP/PTX NLCs, (3) PTX NLCs, (4) DDP NLCs; (2-2) changes in DEE in the presence of serum: (1) DDP DEE of FA-DDP/PTX NLCs, (2) PTX DEE of FA-DDP/PTX NLCs, (3) DDP DEE of DDP/PTX NLCs, (4) PTX DEE of DDP/PTX NLCs, (5) PTX DEE of PTX NLCs, and (6) DDP DEE of DDP NLCs.

**Table 2.** Characterization.

Formulations	NLCs	DDP NLCs	PTX NLCs	DDP/PTX NLCs	FA-DDP/PTX NLCs
Particle size (nm)	96.7 ± 2.6	100.2 ± 3.5	99.8 ± 3.7	101.9 ± 4.8	127.1 ± 5.1
Size distribution (PDI)	0.13 ± 0.03	0.17 ± 0.04	0.19 ± 0.05	0.21 ± 0.06	0.23 ± 0.07
Zeta potential (mV)	31.9 ± 3.4	39.5 ± 3.6	36.7 ± 3.1	43.7 ± 4.1	26.7 ± 2.2
DEE of DDP (%)	N/A	85.6 ± 3.9	N/A	84.9 ± 2.9	82.1 ± 3.4
DEE of PTX (%)	N/A	N/A	80.6 ± 2.8	81.6 ± 3.3	79.2 ± 3.1
DLC of DDP (%)	N/A	13.2 ± 1.2	N/A	9.1 ± 0.9	5.2 ± 0.6
DLC of PTX (%)	N/A	N/A	10.6 ± 1.4	8.3 ± 1.0	4.7 ± 0.5

### Serum stability

Figure 2(1) and (2) describes the changes in size and DEE in the presence of serum. All kinds of formulations were stable up to 24 h without any significant size or DEE changes. FA-DDP/PTX NLCs, DDP/PTX NLCs, DDP NLCs, and PTX NLCs were considered very stable after incubation with FBS and suggest that this formulation will not aggregate or disassemble after intravenous administration.

### *In vitro* drug release

**Figure 3.**
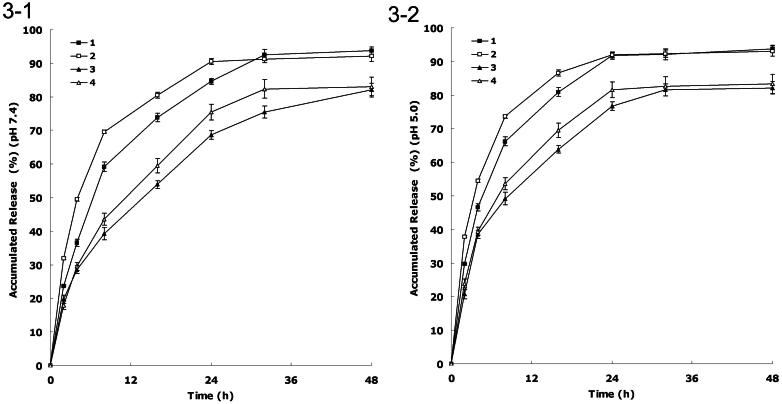
(1) *In vitro* DDP and PTX release at pH 7.4; (2) *in vitro* DDP and PTX release at pH 5.0: (1) DDP releases from FA-DDP/PTX NLCs, (2) DDP release from DDP/PTX NLCs, (3) PTX release from FA-DDP/PTX NLCs, and (4) PTX release from DDP/PTX NLCs.

Figure 3(1) and (2) shows the release curve of DDP and PTX from the NLCs at pH 7.4 and 5.0, respectively. There was a fast release of drugs during the first 8 h, followed by a sustained release in the subsequent 16 h. The releases of drugs from FA decorated NLCs were slower than non-decorated NLCs. The releases of DDP from NLCs were faster than that of PTX. The releases of DDP and PTX in the acidic condition were faster than that of neutral environment.

### Cytotoxicity

**Figure 4.**
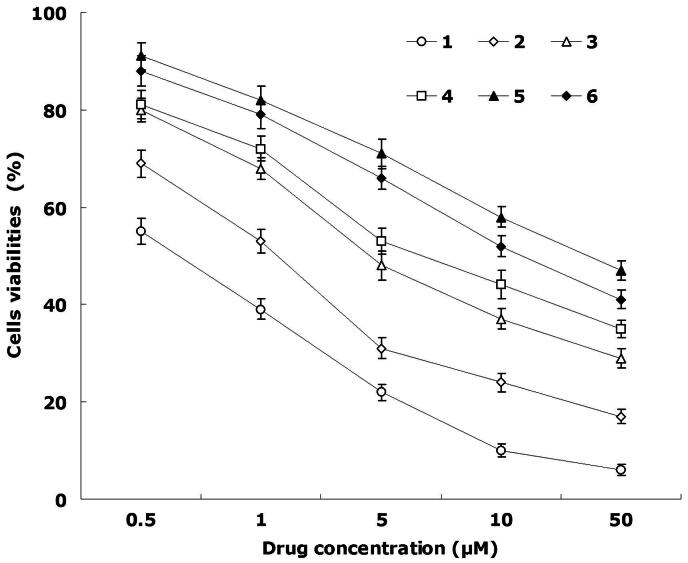
*In vitro* cell viabilities: (1) FA-DDP/PTX NLCs, (2) DDP/PTX NLCs, (3) PTX NLCs, (4) DDP NLCs, (5) PTX solutions, and (6) DDP solutions.

Figure 4 shows the viabilities of cancer cells evaluated by MTT assay. The results illustrated than over the studied drug concentrations, the cytotoxicity of the dual drugs-loaded NLCs were higher than single drug-loaded NLCs (*p* < 0.05); cytotoxicity of the drug loaded NLCs were higher than free drug solutions (*p* < 0.05). The IC_50_ value of FA-DDP/PTX NLCs was the lowest (0.6 ± 0.1 μM), lower than that of DDP/PTX NLCs (1.1 ± 0.3 μM) and other formulations. The IC_50_ values were summarized in Table 3. The IC_50_ values of NLCs formulas were several folds than the free drug solutions. IC_50_ value of FA-DDP/PTX NLCs and DDP/PTX NLCs was 0.57 and 0.81, shown significantly synergism effect. The cell viability of blank NLCs was over 80% at all studied concentrations, showing the safety of the carriers.

**Table 3.** The IC_50_ values and CI_50_.

Formulations	DDP solutions	PTX solutions	DDP NLCs	PTX NLCs	DDP/PTX NLCs	FA-DDP/PTX NLCs
IC_50_ of DDP (μM)	46.5 ± 3.1	N/A	4.7 ± 0.8	N/A	1.1 ± 0.3	0.6 ± 0.1
IC_50_ of PTX (μM)	N/A	36.9 ± 3.6	N/A	6.1 ± 0.7	1.1 ± 0.3	0.6 ± 0.1
CI_50_	N/A	N/A	N/A	N/A	0.81	0.57

### *In vivo* tissue distribution

**Figure 5.**
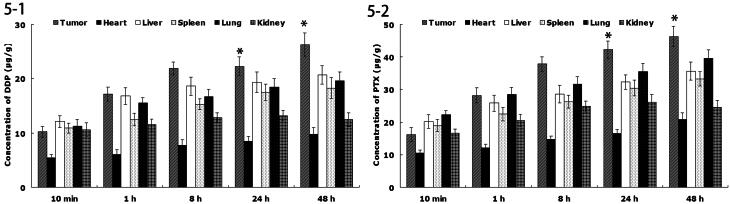
(1) *In vivo* DDP tissue distribution of FA-DDP/PTX NLCs; (2) *in vivo* PTX tissue distribution of FA-DDP/PTX NLCs.

Figure 5(1) and (2) shows the *in vivo* DDP or PTX tissue distribution outcomes of FA-DDP/PTX NLCs. The DDP or PTX concentration in tumor, lung, and liver following injection of FA-DDP/PTX NLCs was higher, in the mean while, the drug concentration in heart and kidney was lower. The drug concentrations of DDP or PTX in the tumor tissue remained relatively stable at all time points until 48 h after injection.

### *In vivo* anticancer efficiency

**Figure 6.**
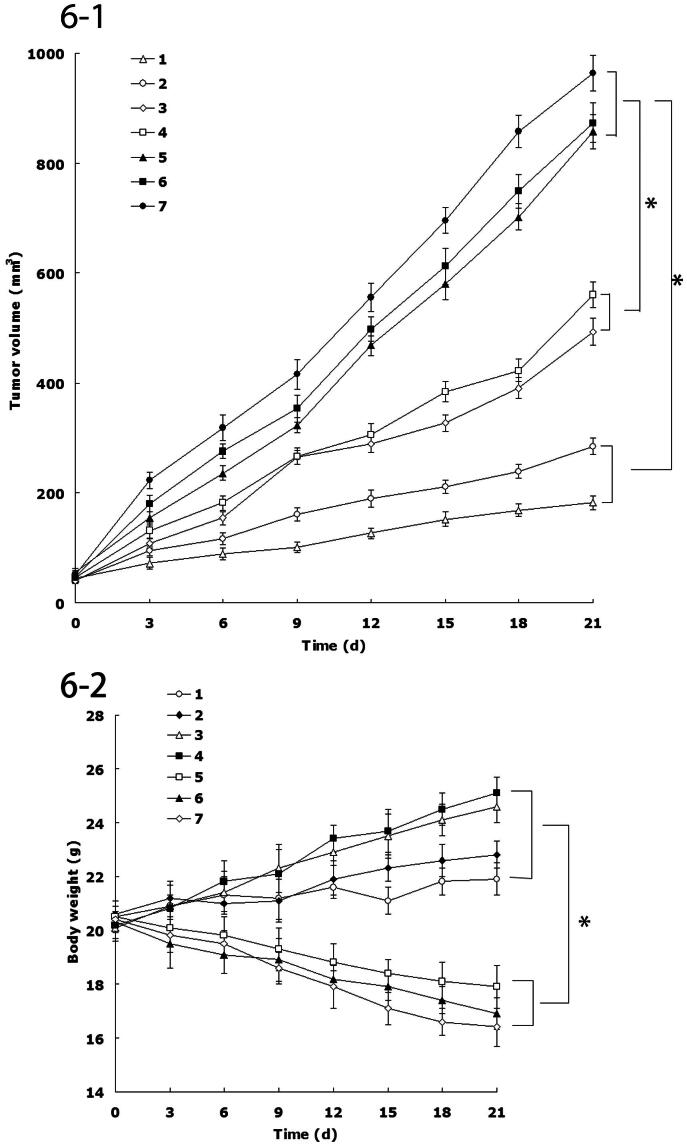
(1) The tumor growth curves; (2) body weight change curves: (1) FA-DDP/PTX NLCs, (2) DDP/PTX NLCs, (3) PTX NLCs, (4) DDP NLCs, (5) PTX solutions, (6) DDP solutions, and (7) 0.9% saline.

The *in vivo* antitumor efficiency was evaluated in the FaDu cells bearing head and neck cancer mice model. As illustrated in Figure 6(1), most obvious tumor regressions were clearly observed in the FA-DDP/PTX NLCs group, the tumor growth was prominently delayed, which attained about 182 mm^3^, whereas in the saline-treated group, tumor volume grew rapidly to 963 mm^3^ on 21-d post-treatment. The TGI of head and neck cancer mice treated with the FA-DDP/PTX NLCs was 81.1%, which significantly higher than that treated with DDP/PTX NLCs and other formulations (Table 4). The obviously emaciation could be observed in the free drug solutions groups, the body weight of free drug groups decreased obviously. However, the NLCs groups did not cause significant difference bogy weight lost (Figure 6(2)).

**Table 4.** The tumor growth inhibition ratio (TGI).

Formulations	DDP solutions	PTX solutions	DDP NLCs	PTX NLCs	DDP/PTX NLCs	FA-DDP/PTX NLCs
TGI (%)	9.3	11.0	41.7	48.8	70.4	81.1

## Discussions

In the present study, DDP/PTX NLCs were prepared by the nanoprecipitation technology. FA-PEG-DSPE was synthesized and modified onto the surface of drugs loaded NLCs. The aim of this research is to achieve good anti-tumor effects on head and neck cancer cells and animal model.

The PEG-DSPE end of FA-PEG-DSPE ligands would live the negatively charged phosphate group was exposed and could readily absorb onto the cationic NLCs surface by charge attraction, and also the lipid end of the ligands could insert into the lipid surface of the carriers by the lipid to lipid affinity (Han et al., 2014; Fang et al., 2016). As the decoration goes on, the negatively charged FA-PEG-DSPE ligands could neutralize the positive surface charge of the NLCs, causing the decrease of the zeta potential. No further decrease of the potential signaled the completion of decoration. On the contrary, overmuch coating of ligands may cause the aggregation of the carriers, leading to a sudden increase in particle size. Table 1 shows the change of size and potential during the decoration process. The decoration ratio was determined as 0.3 g of FA-PEG-DSPE dissolved in 40 ml of PBS to get the FA-PEG-DSPE solution in the preparation process of FA-DDP/PTX NLCs.

The coating of ligands increased the size of the carriers. No significant variations were observed in encapsulation efficiency of both two drugs loading in all kinds of NLCs. These results suggest that a negligible amount of DDP and PTX was leaked during the process of decorating FA-PEG-DSPE to the surface of DDP/PTX NLCs; also the loading of two drugs did not affect the drug loading of one single drug (Wang et al., 2016).

Serum stability is an important index to determine the stability of the NLCs system (Bernkop-Schnürch et al., 2000). All formulations tested were stable up to 24 h without any significant size or DEE changes. The results illustrated that these carriers were considered very stable after incubation with FBS and suggested that this formulation will not aggregate or disassemble after intravenous administration.

*In vitro* drug release of the formulations showed a fast release of drugs during the first a few hours then followed by a sustained release in the subsequent time. The drug release from nanoparticles generally takes place by several mechanisms, including surface and bulk erosion, disintegration, diffusion, or desorption (Rahman et al., 2013). This sustained release of drug from nanoparticles is due to homogenous entrapment of the drug. At high lipid concentration, drugs enriched core can develop that will slow down release of the drugs. Also the surface coating of ligands could slow the release of drug from NLCs. The *in vitro* release profile shows that FA-DDP/PTX NLCs has the capacity to release DDP and PTX at a sustained rate over 48 h, thus let the drugs contentiously develop the therapeutic effect.

*In vitro* cytotoxicity against head and neck cancer efficacy of various kinds of NLCs we evaluated the performance of NLCs in FaDu cells. The results could be explained as follows: first, the construction of NLCs systems could improve the delivery of drugs thus gain better efficiency than free drugs; second, by the combined anti-tumor effect of the dual drugs loaded NLCs than the single drug loaded ones; finally, the surface decoration of dual drugs loaded NLCs using folate containing ligands could have the superiority over the non-decorated drugs carriers and develop the ability of the drugs to a large extent.

*In vivo* drug distribution in heart and kidney may cause systemic toxicity; on the contrary, distribution mainly in the tumor tissue compared with the other tissues could decrease the side effects and lead to better anti-tumor therapeutic efficiency (Ding et al., 2015). Solid tumors have leakage micro vasculatures and the nano-sized particles could passive targeted to the tumor owing to the enhanced permeability and retention (EPR) effects. EPR effects prevented the entry of nanoformulation in the normal cell at the same time favored selective entry in tumor, which resulted in the efficient drug accumulation in tumor tissue (Sun et al., 2016). The drug concentrations in the tumor tissue remained high until 48 h after injection, indicate the sustained-release behavior of the FA-DDP/PTX NLCs. The long circulating effect of NLCs was attributed due to the presence of PEG chain on the surface of particles, which provided stealth effect to the system.

*In vivo* antitumor efficiency was evaluated in the FaDu cells bearing head and neck cancer mice model. Although DDP and PTX are among the most effective anticancer agents against head and neck cancer, it is associated with various systemic side effects, such as nephrotoxicity, myelosuppression, neurotoxicity, nausea, and emesis (Wang et al., 2013). NLCs formulations in this study were used to overcome these side effects and achieve high anticancer efficiency. The tumor inhibition ability of head and neck cancer mice treated with the FA-DDP/PTX NLCs was significantly higher than that treated with DDP/PTX NLCs and other formulations. The obviously emaciation could be observed in the free drug solutions groups, while the NLCs groups did not cause significant difference bogy weight lost. During the treatment, reduction in food intake, energy sag, and inactive in moving were also observed in the free drug solution groups but not in other groups. The results suggested the best anti-tumor effect of folate-decorated double drugs contained NLCs due to the synergetic effect of the two drugs, and the least systemic toxic side effect of the NLC formulations for the head and neck cancer treatment.

## Conclusions

FA-DDP/PTX NLCs were prepared and used for the delivery of DDP and PTX to the head and neck cancer FaDu cells to inhibit the cell viability. Furthermore, the FA-DDP/PTX NLCs effectively improves anticancer efficiency for head and neck cancer-bearing mice without causing obvious toxicity *in vivo*. These results demonstrate that the folate-decorated NLCs may have potential to be used as a targeted molecular medicine for the treatment of head and neck carcinoma.
